# Characterisation of autophagy disruption in the ileum of pigs infected with *Lawsonia intracellularis*

**DOI:** 10.1007/s11259-021-09847-7

**Published:** 2021-10-20

**Authors:** Hamish A. Salvesen, Fiona A. Sargison, Alan L. Archibald, Tahar Ait-Ali

**Affiliations:** The Roslin Institute and Royal (Dick) School of Veterinary Studies, University of Edinburgh, Easter Bush, Edinburgh, UK

**Keywords:** Autophagy, Lawsonia intracellularis, Pig, Proliferative enteropathy, Intestinal homeostasis

## Abstract

*Lawsonia intracellularis* is the aetiological agent of proliferative enteropathy, an enteric disease endemic in swine. Survival in its intracellular niche of the ileum epithelial lining requires the capacity to subvert, repress or exploit the host immune response to create an environment conducive to bacterial propagation. To better understand how *L. intracellularis* survives in its intracellular niche, we have performed an investigation into the dynamic relationship between infection and the host autophagy response by immunohistochemistry in experimentally infected porcine ileum samples.

Beclin1, a protein required early in the autophagy pathway was observed to be distributed with a basal to apical concentration gradient in the crypts of healthy piglets, whilst infected piglets were observed to have no gradient of distribution and an increase in the presence of Beclin1 in crypts with histological characteristics of *L. intracellularis* residence. Detecting microtubule-associated protein light chain 3 (LC3) is used as a method for monitoring autophagy progression as it associates with mature autophagosomes. For LC3 there was no notable change in signal intensity between crypts with characteristic *L. intracellularis* infection and healthy crypts of uninfected pigs. Finally, as p62 is degraded with the internal substrate of an autophagosome it was used to measure autophagic flux. There was no observed reduction or redistribution of p62.

These preliminary results of the autophagy response in the ileum suggest that *L. intracellularis* affects autophagy. This disruption to host ileum homeostasis may provide a mechanism that assists in bacterial propagation and contributes to pathogenesis.

Proliferative Enteropathy (PE) is principally a porcine disease with clinical manifestations most commonly observed in juveniles post-weaning (Lawson and Gebhart 2000). Mild to severe diarrhoea, intestinal adenomas and macroscopically obvious thickening of the intestinal mucosa, result in anorexia, poor feed conversion and growth rates stunted by as much as 50% (Lawson and Gebhart 2000). As infections are often subclinical and complicated to confirm, it is likely the impact of PE is broader than epidemiological and clinical data currently suggest (Brandt et al. 2010).

*Lawsonia intracellularis* is a Gram-negative, obligate intracellular bacterium with a tropism for the apical region of enterocytes of the intestinal ileum (McOrist et al. 1995). *L. intracellularis* infection impedes the maturation of transit amplifying cells, which remain in a proliferative state whilst migrating up the villi (McOrist et al. 1992). This maintenance of proliferative state is also relevant to the loss of goblet cells and the associated mucous layer they secrete (Huan et al. 2017). It is unknown whether the hyperplastic response and observed changes in host gene expression are host-induced to promote the clearing of *L. intracellularis* by cellular shedding, or bacterial manipulation of host molecular pathways to increase the proportion of hospitable host cells and transmission (Vannucci et al. 2013; Leite et al. 2019).

Macroautophagy is a highly conserved eukaryotic process whereby cytosolic substrates are sequestered and delivered to lysosomes for degradation (Mizushima 2007). Autophagy also functions as part of the innate immune response against intracellular pathogens. The steps of autophagy include initiation, substrate targeting, elongation and maturation/lysosomal fusion (Kimmey and Stallings 2016). Intracellular pathogens must either avoid recognition, escape following sequestration, or exploit autophagosome maturation to generate a protective membrane-bound organelle in which to replicate (Cemma and Brumell 2012).

As an obligate intracellular bacterium, a finely balanced relationship with the host environment is required for successful completion of the lifecycle and transmission. No membrane bound vacuoles have been observed in *L. intracellularis-*infected cells, suggesting *L. intracellularis* replicate freely in the host-cell cytosol (McOrist et al. 1995; Lawson and Gebhart 2000). The aim of this study was to provide insight into *L. intracellularis* and its relationship with autophagy. Interpretation of the presence and localisation of autophagy pathway proteins will assist in understanding the pathogenic progression of PE and improve our understanding of how *L. intracellularis* survives and establishes its intracellular niche.

## Materials & methods

### Tissue samples

Porcine ileum samples used for immunohistochemistry analysis were acquired from a set of paraffin blocks obtained from a previous *L. intracellularis* challenge trial for a vaccination study. The animal trial was conducted by the Swine Services Unlimited (MN, USA) (Roerink et al. 2018). Because only paraffin blocks were used institutional ethics committee approval was deemed unnecessary for this current work.

Briefly, paraffin blocks from ileum tissues of pigs 21 days post challenge and infection were empirically assessed by staining for the presence of *L. intracellularis* with VPM53 antibody (University of Edinburgh) and quantitation of fluorescence. The two samples selected for this study were two of the most highly infected available piglets from Roerink et al, 2018 (Roerink et al. 2018). All samples were from the same randomised and blinded vaccination study in a mixed-breed herd that was clinically and ELISA negative for *L. intracellularis.*

### Immunohistochemistry

Tissue Sects. (4 µM thick) used were from sequential ileum. From the uninfected and infected cohorts 2 pigs were selected for investigation for all staining experiments, excepting LC3 which had only a single control pig stained. Except for *L. intracellularis* (VPM53) staining, all slides underwent heat-mediated antigen retrieval in citrate buffer, pH6, using a Histo5 Rapid Microwave Histoprocessor (Milestone Medical) under high pressure at 110 °C for 5 min. For VPM53 tissue samples were incubated in proteinase K (DAKO UK Ltd.) for 20 min at room temperature followed by two washes in phosphate buffered saline (PBS). Cells were permeabilised with 0.1% Triton-X (Sigma-Aldrich) in PBS for 10 min at room temperature followed by two further PBS washes. Samples were blocked with 5% skim milk powder (SMP) in PBS at room temperature for 1 h, before incubation at 4 °C overnight with the primary antibody (Beclin1 (Abcam;ab231341), LC3 (Cell Signalling Technology;CST3868) and p62 (Abcam;ab101266)) in 5% SMP in PBS. Slides were washed in PBS before incubation with secondary antibodies (VPM53;Alexa-Fluor 488 goat anti-mouse, all others Alexa-Fluor 647 goat anti-rabbit, ThermoFisher Scientific) at a concentration of 1:1000 for 1 h at room temperature. Samples were counter-stained with 6-diamidino-2-phenylindone (DAPI) before a coverslip was mounted with LabVision PermaFluor (ThermoFisher Scientific).

### Intestinal alkaline phosphatase stain

Intestinal alkaline phosphatase (IAP) was monitored with Vector® Red Alkaline Phosphatase Substrate (Vector Laboratories) according to manufacturer instructions (Roerink et al. 2018). Slides were stained with DAPI and mounted as described above. The presence of intestinal alkaline phosphatase was viewed with a Texas Red® filter using a Leica DMLB Fluorescence microscope.

### Microscopy and quantitative image analysis

Autophagy markers were visualised at 20 × magnification using a Zeiss Laser Scanning Microscope 710 (Carl Zeiss) and imaged using Zen Black Software (Carl Zeiss). Only regions of the ileum sectioned to show entire longitudinal crypts were selected in the optical field for imaging. Because *L. intracellularis* is highly localised, crypts displaying a proliferative state indicative of *L. intracellularis* infection were selected for imaging. All images and all crypts in the images were quantified. For measuring the expression gradient of crypts, only entire longitudinal crypts were quantified. To remove background noise in imaging, gain values were set on a negative control incubated with only the secondary antibody and DAPI. These gain and exposure time settings were consistent for each experiment.

Fluorescence intensity was quantified using FIJI (Fiji Is Just ImageJ, U.S. National Institutes of Health, USA). Samples from 2 uninfected and 2 experimentally infected piglets were used for each condition, with at least 9 discrete regions of the ileum imaged per section. Regions of interest (ROI) were manually outlined, the spectrally opposite colour of interest was selected, and global thresholding was set at 60 & 255 to highlight the marker and account for background fluorescence. For crypts and epithelial lining measurements, signal intensity was normalised to the area of the entire field. Villus epithelial lining was selected using the line tool on a thickness of ten. The mean area:signal ratio of the ROI as determined using ImageJ was used to consider crypt size and localisation of proteins.

## Results

### Assessing proliferative enteropathy following *L. intracellularis* infection

**Fig. 1:**
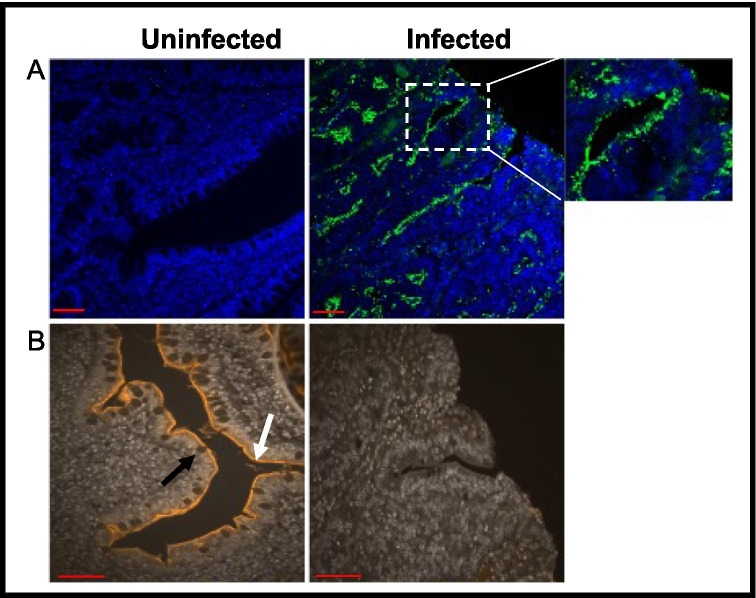
Images of ileum samples from uninfected and infected piglets illustrating *Lawsonia intracellularis* infection and classical PE progression. Scale bars represent 10 μm A) IHC demonstrating *L. intracellularis* (green fluorescence; VPM53) residing specifically in the apical region of intestinal epithelia. B) Intestinal Alkaline Phosphatase (IAP) staining. The white arrow indicates the IAP lining, and the black arrow indicates a goblet cell

To establish the samples as appropriate for autophagy investigation we first investigated the pathohistology of ileum samples from uninfected and *L. intracellularis-*infected piglets. The presence of *L. intracellularis* in epithelial cells in the ileum was confirmed in the infected samples (Fig. 1A) and the level of infection in the pigs that were experimentally exposed to *L. intracellualris* was previously categorised by Roerink et al. 2018.

The disruption of the intestinal topology and hyperplasia of epithelial cells is characteristic of proliferative enteropathy (McOrist et al. 1996). Staining for IAP showed a severe loss of the mucin layer coating epithelial tissue of the ileum in infected piglets and inflammation causing the loss of the finger-like villi (Fig. 1B) (Fawley and Gourlay 2016). The absence of goblet cells from the inflamed regions was notable as goblet cells produce the protective mucin layer (Specian and Oliver 1991).

### Autophagy progression during proliferative enteropathy

Markers of key steps in the autophagy pathway were examined by immunohistochemistry in infected and uninfected samples. The measurement of fluorescence indicates the quantity of the protein of interest in a specified area, an increased mean fluorescence signal is therefore interpreted as an increase in protein in the region being measured.

**Fig. 2:**
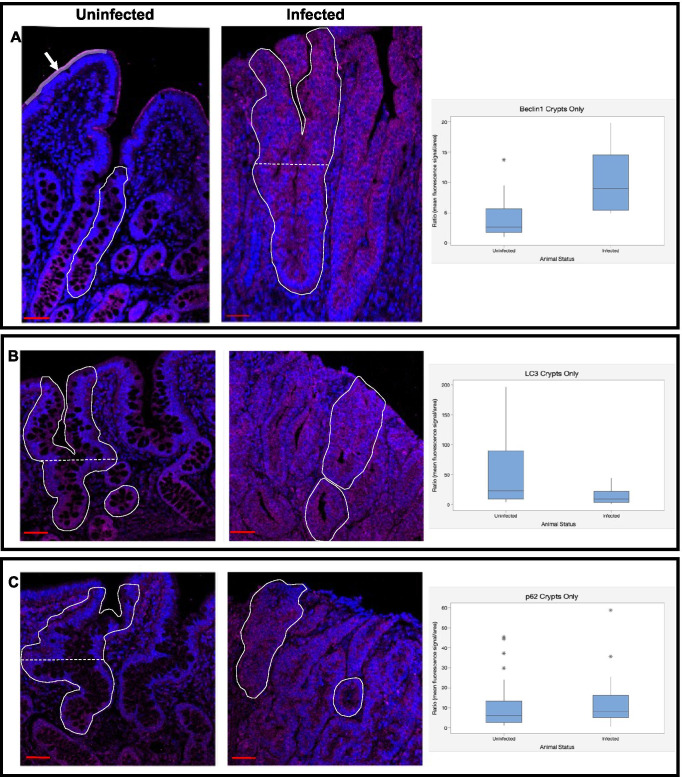
Representative images demonstrating the distribution and presence of autophagy pathway proteins Beclin1, LC3 and p62 in piglet ileum sections*.* All images are at 20 × magnification. Cells were counterstained with DAPI (Blue). Fluorescence values were calculated by mean signal intensity over the area measured. Scale bars represent 10 μm. The white enclosing lines in A indicate example regions measured for mean fluorescence signal for full longitudinal crypts. The horizontal line is indicative of the half-way point in a crypt used to compare fluorescence intensity in the apical versus basal regions for quantification of the expression gradient. Asterisks (*) denote outliers, measurements that were greater than 1.5 times the length of the box from the nearest box end. **A**). A representative image of Beclin1 (purple) from an uninfected and infected sample and quantification of the presence in crypts in entire longitudinal crypts. Beclin1 was observed as a defined epithelial layer on villi tips. The area beneath the grey line (white arrow, top left) on the epithelial lining of the uninfected sample image is an example of the area quantified for fluorescence intensity on the epithelial lining. **B**) A representative image of LC3 (purple) staining in an uninfected and infected piglet and quantification of LC3 presence in entire longitudinal crypts. **C**) A representative image of p62 (purple) in an uninfected and infected sample and quantification of p62 presence in entire longitudinal crypts

Beclin1 was selected as it is an almost ubiquitous component of mammalian autophagy initiation. Its wider functional roles associated with differentiation were also of interest due to the disruption of homeostasis that is observed with PE (Cao and Klionsky 2007). Changes in the presence and distribution of Beclin1 were observed in tissues from the infected samples (Table 1, Fig. 2A). The fluorescence intensity was observed to increase in crypt regions (Fig. 2A). In uninfected pigs there was a basal to apical Beclin1 gradient observed, this was perturbed and became evenly distributed through the crypts showing characteristics of *L. intracellularis* residence in the infected pig samples. On the epithelial lining of villi in uninfected piglets, accumulation of Beclin1 was observed on the brush border in a manner similar to that seen with IAP. Crypts displaying a hyperplastic response in the infected samples were observed to have a reduction of the intestinal brush border signal of Beclin1.

**Table 1:** Descriptive statistics of ileum fluorescence measurements

Ileum ROI	Infection state	Protein	*n*	Observations	Mean signal	SD	SE
Full	Uninfected	Beclin1	2	14	0.55	0.36	0.1
Full	Uninfected	LC3	1	5	1.04	0.29	0.13
Full	Uninfected	p62	2	9	0.75	0.47	0.16
Crypts	Uninfected	Beclin1	2	16	4.17	3.49	0.87
Crypts	Uninfected	LC3	1	23	55.2	61.7	12.86
Crypts	Uninfected	p62	2	30	11.18	12.83	2.34
Gradient	Uninfected	Beclin1	2	14	3.74	1.89	0.50
Gradient	Uninfected	LC3	1	12	1.81	0.77	0.22
Gradient	Uninfected	p62	2	20	1.18	0.7	0.15
Full	Infected	Beclin1	2	9	1.05	0.21	0.07
Full	Infected	LC3	2	12	0.64	0.07	0.25
Full	Infected	p62	2	10	1.05	0.6	0.19
Crypts	Infected	Beclin1	2	19	9.97	5.03	1.15
Crypts	Infected	LC3	2	46	13.5	11.43	1.69
Crypts	Infected	p62	2	36	12.02	10.96	1.83
Gradient	Infected	Beclin1	2	24	1.39	0.27	0.05
Gradient	Infected	LC3	2	23	1.02	0.49	0.1
Gradient	Infected	p62	2	23	1.01	0.39	0.08

Cytosolic microtubule-associated protein light chain 3 (LC3) is conjugated to phosphatidylethanolamine to form LC3-II before joining the autophagosome as the penultimate protein for maturation. Cytosolic puncta of LC3-II are therefore utilised as a marker for mature autophagosome formation (He et al. 2015). The presence and distribution of LC3 was investigated to examine the formation of autophagosomes in *L. intracellularis-*infected piglet ileums. The level of LC3 remained similar following infection (Table 1, Fig. 2B). Uninfected pigs had a basal to apical crypt gradient of LC3, whereas in infected piglets LC3 became distributed consistently throughout crypts displaying characteristics of *L. intracellularis* infection (Fig. 2B).

p62 contains a ubiquitin binding domain and a domain that binds specifically to the LC3-II isoform, thus acting as a mediator between the ubiquitin detection innate immune response and the formation of the mature autophagosome (Pankiv et al. 2007). Following lysosomal fusion, p62 is degraded along with the sequestered internal cargo. In samples from infected pigs, crypts displaying characteristic residence of *L. intracellularis* had no observable difference in distribution of p62 when compared to the uninfected piglets (Table 1, Fig. 2C). Given that observations of p62 can be as a method for measuring the completion of autophagy through its degradation, this result suggests that the full cycle of autophagy is not increased in *L. intracellularis* infected crypts of infected pigs compared to uninfected pigs (Pankiv et al. 2007).

## Discussion

As an intracellular pathogen, *L. intracellularis* is in a constant arms race with its unobliging host to create a niche conducive to its replication and survival. It had been suggested earlier that *L. intracellularis* did not exploit the autophagosome as an intracellular niche, and thus a mechanism of escape or repression of autophagy was likely to exist (McOrist et al. 1995; Lawson and Gebhart 2000). Our analyses are consistent with this suggestion, further adding that early autophagic steps proceed and contribute to a reduction in differentiation and maintain a proliferative state in the niche of *L. intracellularis*. This limited data set provides preliminary data to suggest that the relationship between *L. intracellularis* and autophagy could be contributing to the clinical manifestation and transmission of PE.

### The autophagy response

Using immunohistochemistry, we investigated the distribution of autophagy pathway proteins in ileum samples infected with *L. intracellularis* to further understand how it survives and replicates in its intracellular niche of epithelial cells in the ileum. Beclin1 had a distinct gradient in uninfected samples, with levels decreasing going up the villi. Although less pronounced, the LC3 gradient augments the Beclin1 observations of autophagy occurring at a higher level closer to the base in crypts of uninfected porcine ileum, augmenting results previously reported in the murine jejunum (Asano et al. 2017).

As a mediating protein between ubiquitin and LC3, the degradation of p62 can be used as a proxy for autophagy turnover (Pankiv et al. 2007). The lack of p62 and LC3 accumulation or puncta leads to the suggestion that *L. intracellularis* may evade autophagy before p62 inclusion in the pathway and that *L. intracellularis* does not rely on the maturation of an autophagy-associated vacuole to support survival and replication. The methods required with our VPM53 antibody for *L. intracellularis* are unfortunately not amenable to being used concurrently with other antibodies, meaning co-localisation of *L. intracellularis* with the autophagy markers was unable to confirmed in this study, however the distinctive inflammation and loss of cell differentiation characteristic of PE was used to determine *L. intracelllularis* infected regions.

### Intestinal topology

**Fig. 3:**
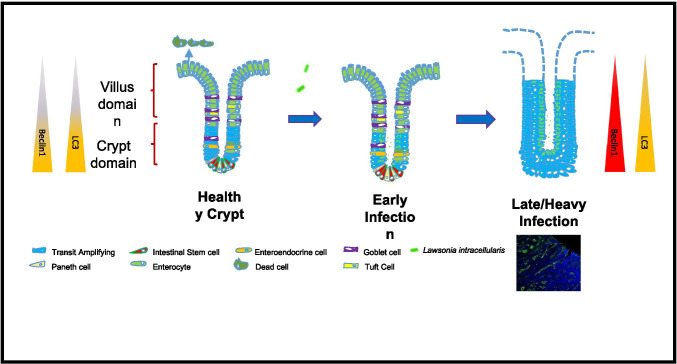
A schematic representation of the response of *L. intracellularis-*infected ileum. Uninfected porcine ileum contains diverse cell types that differentiate as they migrate up villi and shed at the apex. Infected cells enter a hyperplastic state, retaining an immature transit amplifying status and reducing the level of cellular shedding. Beclin1 and LC3 have a distinct basal to apical gradient in uninfected porcine ileum crypts which becomes distributed throughout the ileum following infection

Autophagy is recognised as being critical to the regulation of the cell cycle. It regulates cellular proliferation, which is the principal driving force of cell migration from crypts to villi and intestinal stem cell differentiation (Parker et al. 2017). Beclin1 and LC3 contribute to intestinal epistasis through inhibition of cellular differentiation and stimulation of proliferation (Wu et al. 2016). Our observations of autophagy related proteins being distributed in gradient in uninfected porcine crypts further supports the suggestions that autophagy has a role in regulating intestinal topology and maintaining the regenerative dynamic of epithelial cell migration (Fig. 3). The hyperplastic state observed in ileums of pigs with PE may therefore be associated with increased Beclin1.

It should be taken into account that the number of cells present in hyperplastic regions will be far greater than in healthy crypts, and therefore the increase in cell number could be accounting for some of the increase in signal intensity detected. However, as only Beclin1 was observed to increase in signal intensity, either the presence of LC3 and p62 is reduced relative to this cell number increase and Beclin1 remains more stable, or Beclin1 increases and LC3 and p62 do not. This consideration emphasises the need for future work to be performed with co-localisation of autophagy markers and *L. intracellularis*.

With a contiguous presence on the brush border of healthy porcine villi, Beclin1 may have a role in early detection of bacterial invasion, orchestrating the autophagic response against bacteria capable of penetrating the mucous lining (Benjamin et al. 2013). The distribution and expression level of Beclin1 may be associated with the loss of the protective mucous membrane observed with PE and *L. intracellularis* invasion, however more investigations are required to better understand the molecular mechanisms at play.

The results from this study have made initial steps into characterising the molecular interactions between the innate immune response of autophagy and *L. intracellularis* during PE*.* These novel insights into *L. intracellularis* and autophagy provides a framework from which to begin future studies, progressing towards the overarching goal of developing more effective control methods of *L. intracellularis* to reduce the burden of PE in swine herds and the financial impact it has on farmers.

## Data Availability

The data analysed in this investigation are available upon request to the corresponding author.
